# Biosorption of cesium and strontium from aqueous solution by *Aspergillus flavus* biomass

**DOI:** 10.1038/s41598-025-11603-9

**Published:** 2025-07-20

**Authors:** A. M. Mousa, Mohamed M.E. Breky, M. F. Attallah

**Affiliations:** 1 Soil and Water Research Department, Nuclear Research Center,, Egyptian Atomic Energy Authority (EAEA), Abu Zaabal, Cairo, 13759 Egypt; 2 Radiation Protection and Safety Department, Hot Laboratories and Waste Management Center, , Egyptian Atomic Energy Authority (EAEA), 13759 Abu Zaabal, Cairo, Egypt; 3 Analytical Chemistry and Control Department, Hot Laboratories and Waste Management Center, , Egyptian Atomic Energy Authority (EAEA), 13759 Abu Zaabal, Cairo, Egypt

**Keywords:** *Cesium*, *Adsorption*, *Radioisotope*, *Strontium*, *Radioactive liquid waste*, Biotechnology, Chemistry, Materials science

## Abstract

This study investigates the potential biosorption of *Aspergillus flavus* biomass for the removal of Cs⁺ and Sr²⁺ ions from aqueous solutions. The biosorbent was characterized using FTIR, SEM-EDS, BET surface area analysis, and thermal stability tests, revealing key functional groups (–OH, –COOH, –NH₂) and a surface area of 9.65 m²/g. Batch adsorption experiments demonstrated that pH significantly influenced uptake, with optimal removal at pH 5 for Sr²⁺ (~ 90%) and pH 8 for Cs⁺ (~ 27%). Kinetic studies followed the pseudo-second-order model (R² > 0.97), indicating chemisorption dominance. Equilibrium data fitted the Freundlich isotherm, suggesting multilayer adsorption, with maximum capacities (qₘₐₓ) of 211.1 mg⋅g^−1^ (Sr²⁺) and 26.7 mg⋅g^−1^ (Cs⁺). Thermodynamic analysis revealed endothermic (ΔH > 0), spontaneous (ΔG < 0), and entropy-driven (ΔS > 0) adsorption. Competitive ion studies showed Ca²⁺ strongly inhibited Sr²⁺ uptake, while Na⁺ reduced Cs⁺ adsorption. The biosorbent exhibited excellent reusability (3 cycles) with 0.1 M HNO_3_ as the best eluent (81.2% Sr²⁺, 71.5% Cs⁺ recovery). The proposed mechanisms include ion exchange, surface complexation, and electrostatic interactions. These findings highlight *A. flavus* as a promising, low-cost biosorbent for nuclear wastewater treatment.

## Introduction

Various nuclear facilities generate radioactive waste effluents with varying compositions and quantities globally^1^. Among these wastes, isotopes such as Cs, Eu, Co, and Sr emerge as primary components, particularly in radioactive liquid waste. Due to their long half-lives (e.g., ¹³⁴Cs: 2.06 years; ¹³⁷Cs: 30.17 years; ⁹⁰Sr: 28.79 years) and interactions with water/soil, these isotopes pose significant environmental risks, especially following nuclear accidents, tests, and inadvertent leaks^2–4^. Long-lived isotopes like ¹³⁷Cs and ⁹⁰Sr accumulate in soil, plants, and water, posing persistent ecological threats^2,5^. The nuclear industry faces persistent challenges in treating wastewater contaminated with ¹³⁷Cs and ⁹⁰Sr, particularly after the 2011 Fukushima incident, which released large volumes of radioactive effluent^6^. Both radionuclides are highly soluble, which contributes to their mobility and persistence in the environment. ^137^Cs, in particular, often dominates the radioactive activity in nuclear waste^7^. The hazards associated with radionuclides in water pose severe risks to human health, particularly from radioactive fallout linked to nuclear plants and operations^8^. Additionally, global concern over heavy metal pollution is increasing, as these metals enter the environment through natural processes (e.g., volcanic eruptions, forest fires) and human activities (e.g., mining, industrial production)^9^. Treating wastewater containing radionuclides remains one of the nuclear industry’s most pressing challenges.

Cs^+^ is employed in photoelectric cells, vacuum tubes, and catalytic applications. The isotope ¹³⁷Cs, a gamma emitter, is used in radiotherapy and as a calibration source for radiation detectors^10^. Exposure to this radionuclide may induce cellular damage, increasing carcinogenic risk. Natural zeolites effectively adsorb Cs⁺ ions from aqueous solutions^11^. Sr compounds find applications in glass, ceramics, pyrotechnics, and palliative care for bone cancer pain. Due to its chemical similarity to calcium, Sr^2+^ can incorporate into bone tissue, potentially causing skeletal disorders.

Remediation of radioactive contaminants can be achieved through various methods, including micro-remediation^12^microbe-aided phytoremediation^13^ nano-bioremediation^14 ^and systems biology^15^. Methods such as nanofiltration and reverse osmosis are proficient at eliminating multivalent ions, including Sr²⁺. Electrodialysis and membrane distillation have demonstrated potential, particularly in the treatment of radioactive wastewater^16^. Thermally treated natural zeolites exhibit improved adsorption capabilities^17^. Porous zirconium phosphate and layered metal sulfides exhibit remarkable efficiency and selectivity in the removal of Sr²⁺ from wastewater^18^.

Fungi, a diverse group of eukaryotic microorganisms (including molds, yeasts, and mushrooms), exhibit significant potential in biosorption due to their unique physical and biological characteristics^19–22^. Unlike living biomass, Dead fungal biomass offers additional advantages, including environmental resistance, toxicity tolerance, ease of regeneration, and efficient metal recovery^23–26^. The fungal cell membrane consists of a lipid bilayer (40% phospholipids and sterols) and proteins (60%), while the cell wall comprises polysaccharides (e.g., chitin, glucans), proteins, and lipids (80–90% of dry weight)^21,23,27^. These components contain functional groups (e.g., hydroxyl, carboxyl, amine, phosphonate) that bind metal ions^28,29^. Mechanisms of sorption in dead biomass include physical adsorption, ion exchange, chelation, and metal complexation^27^. Fungi demonstrate remarkable metabolic plasticity and polyextremotolerance, enabling survival in harsh environments, including those with low pH and high metal concentrations^22^. Their high cell wall-to-biomass ratio enhances metal-binding capacity, making them superior to other biosorbents^21,30^.

Fungal biomass, including *Aspergillus niger* and *Rhizopus arrhizus*, is a cost-effective and abundant alternative for removing Cs^+^ and Sr^2+^ from radioactive wastewater. These biosorbents have high surface area, efficient binding mechanisms, excellent selectivity, and perform well across various pH conditions^31,32^. They are biodegradable, environmentally friendly, and can achieve comparable or superior removal efficiencies compared to traditional adsorbents^31,32^. Sumalatha et al. (2022) found that agricultural waste-derived materials can effectively remove radionuclides, with 99% Sr²⁺ uptake achieved using KOH-activated peanut shell biochar (PSABC). Fungal biomass like *Aspergillus flavus* was also evaluated for simultaneous Cs⁺/Sr²⁺ removal using advanced kinetic/isotherm modeling^33^. Dulla et al. (2020)^34^ demonstrated that spent *Gelidiella acerosa* biomass achieved 96.4% Cu (II) removal via chemisorption. Khan et al. (2022)^35^ discovered that algal biosorbents like *Nostoc sp.* and *Turbinaria vulgaris* effectively remove heavy metals from industrial effluents, with 94.2% and 88.9% removal achieved under optimized acidic conditions. Recent studies reveal that *Turbinaria vulgaris* can effectively remove arsenic up to 92.12% efficiently under optimized conditions^36^.

*Aspergillus flavus* is a promising biosorbent for radionuclide removal due to its cell wall composition and high surface-area-to-volume ratio. Its dead biomass utility, scalability, and competitive biosorption of Cs⁺ and Sr²⁺ offer new biosorption concepts and insights into selective radionuclide recovery. *Aspergillus flavus* is a sustainable, eco-friendly sorbent that can be safely incinerated or vitrified, offering a promising alternative to synthetic alternatives in the circular economy.

This study investigates the potential of *Aspergillus flavus* biomass as a green method for the removal of Cs^+^ and Sr^2+^ ions (model long-lived fission products) from aqueous solutions. The parameters of the batch biosorption process, including ion concentration, solution pH, shaking time, and temperature, were also evaluated.

## Materials and methods

### Fungal strain and cultivation

The fungus *Aspergillus flavus* was identified and sourced from the soil microbiology Laboratory of the Soil and Water Research Department at the Egyptian Atomic Energy Authority (EAEA). The isolation and identification of *Aspergillus flavus* have been done as shown in a previous study^37^. The fungus was regularly maintained on Czapek-Dox’s agar medium^38^ containing: 20 g of sucrose, 2.0 g of NaNO_3_, 1.0 g of KH_2_PO_4_, 0.5 g of MgSO_4_, 0.5 g of KCl, 0.001 g of FeSO_4_, 0.001 g of CaCl_2_, agar 20 g and distilled water 1 L.

### **Production of*Aspergillus flavus*biomass**

*Aspergillus flavus* was inoculated on flasks containing broth Dox medium. The flasks were then agitated on a horizontal orbital shaker at 25 °C at 150 rpm for 7 days. Harvested mycelium was autoclaved to obtain dead fungal biomass, filtered off, and washed with double-distilled water. Then, it was dried using the lyophilization technique and ground for subsequent use.

### Instruments

The Fourier transform infrared spectra (FTIR) of fungal biomass were kept in the scope of 400–4000 cm^−1^ with an Agilent Cary 630 FTIR spectrometer (Agilent Technologies Inc., Santa Clara, CA, USA). The thermal gravimetric analysis (TGA) of fungal biomass was performed using the Shimadzu DTG-60 thermal analyzer purchased from Shimadzu Kyoto, Japan. The platinum sample holder was used in the experiments. The experiment was conducted in a dynamic oxygen atmosphere with a heating rate of 20 °C/min and temperatures ranging from room temperature to 600 °C. The Brunauer–Emmett–Teller (BET) surface area of fungal biomass was determined using the Nova 3200 series (USA). The scanning electron microscope with energy dispersive spectroscopy (SEM/EDS) analyses was performed with JEOL JSM-IT200 associated with an EDS detector, Japan. It is used to estimate the elemental composition to render a complete knowledge of the simplicity, distribution, and position of the fungal biomass. The investigated ions Cs^+^ and Sr^2+^ were determined using an atomic absorption spectrophotometer, 210VGP.

### Factors controlling the biosorption process

Experiments utilized stable Cs⁺ (as CsCl) and Sr²⁺, (as Sr(NO₃)₂) analogs to simulate ¹³⁷Cs and ⁹⁰Sr behavior, avoiding radiation hazards while maintaining identical chemical properties. All biosorption procedures were conducted using a mixture of Cs^+^ and Sr^2+^ ions in one solution. Several pH levels from 1 to 8 were assessed to determine the influence of pH on the batch sorption process. The experiment was performed under controlled conditions with 0.02 g of dried biomass; 50 mg/L of each ion, Cs^+,^ and Sr^2+^, was agitated on an orbital shaker at 125 rpm and 25 ± 1 °C. The influence of contact time on the biosorption process was investigated at 0 to 60 min intervals for a mixed solution of Cs^+^ and Sr^2+^ (50 mg/L of each ion) at pH 5 and a temperature of 25 ± 1 °C. The impact of the initial concentrations of Cs^+^ and Sr^2+^ on the biosorption process was evaluated at optimum conditions, within the 100 to 1000 mg/L range. The optimal pH and contact time values were obtained from the studies above using 0.02 g of dry biomass as a control experiment. The uncertainty of all measured data did not surpass ± 5%. The subsequent formulas were employed to ascertain the equilibrium absorption of dried biomass and the proportion of metal ion removal:

1 $${q_e}={\text{ }}\left( {{C_i}--{\text{ }}{C_e}} \right){\text{ }}V/m$$

2 $${\text{R }}\left( {\text{\% }} \right){\text{ = }}\frac{{{C_i} - {C_e}}}{{{C_i}}}{{ \times 100}}$$.

Where q_e_ represents the equilibrium sorption of the dried *Aspergillus flavus*, mg⋅g^−1^, C_i_ is the initial concentration (mg/L); C_e_ is the final concentration (mg/L); m is the dried mass of the *Aspergillus flavus* in the reaction (g); V is the volume of the reaction mixture (L), R is the removal %.

### Desorption and reusability of adsorbed metals

To determine which desorbing agents were most effective at removing Cs^+^ and Sr^+2^ ions from the *Aspergillus flavus*, they were independently tested. The desorbing solutions utilized were EDTA (0.1 mol/L), HNO_3_ (0.1 mol/L), H_2_SO_4_ (0.1 mol/L), NaOH (0.1 mol/L), and HCl (0.1 mol/L). In each experiment, 0.02 g of *Aspergillus flavus* was used for 100 ppm of Cs^+^ and Sr^2+^, and 10 mL of the desorbing solution was placed in an Erlenmeyer flask and shaken for 90 min at room temperature. The reusability of the dried biomass was evaluated through several adsorption and desorption cycles. The desorption procedure utilized an optimal concentration of desorbing agents for Cs^+^ and Sr^2+^, with adsorption efficiency assessed after each cycle by the Eq. 

3 $$\% Desorption=\frac{{{\text{Amount of metal ion desorbed}}}}{{{\text{Amount of metal ion adsorbed}}}} \times 100$$.

## Results and discussion

### Characteristics of *Aspergillus flavus*

#### FTIR analysis

**Fig. 1:**
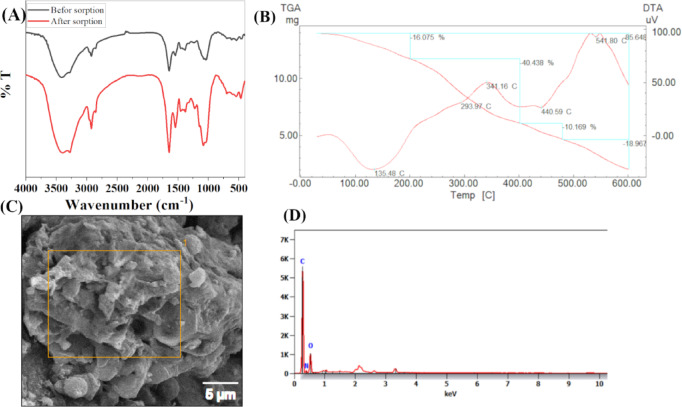
(**A**) FT-IR spectrum, (**B**) Thermal analysis, (**C**) SEM, and (**D**) EDS chart of the *Aspergillus flavus* biomass.

FTIR spectra were recorded to investigate the surface functional groups present in *Aspergillus flavus* biomass before and after biosorption of Cs^+^ and Sr^2+^. A comparative analysis reveals changes in specific functional group vibrations, indicating possible interactions or binding mechanisms between the biomass and the metal ions. The results also indicated that the bands at 3414, 2925, 1646, 1543, 1384, 1295, 1040, 869, 618, 540, and 452 were shifted to 3396, 3279, 2924, 2854, 1646, 1543, 1455, 1380, 1226, 1083, 697, 543 and 468 after sorption of Cs^+^ and Sr^2+^ ions and intensity changes of some peaks compared to the unloaded biomass. The FTIR spectra of the biomass before sorption of Cs^+^ and Sr^2+^ ions are illustrated in Fig. 1**(A)**. Absorption at 3200–3400 cm^−1^ can be correlated to the O–H, –NH group stretch^1,39^. 2925 cm^−1^ is considered to be the stretching vibrations of –CH– in biomass^40^ while 1646 cm^−1^ corresponds to the binding vibrations of the C = C stretching double bond. C = O stretching implies that proteins play a key role in metal binding, likely through the –NH and –C = O groups. The broadening of the –OH/NH peak suggests hydrogen bonding alterations or direct interaction of hydroxyl groups with Cs⁺ and Sr²⁺. Absorption at 1000–1200 cm^−1^ can be correlated to the C–O/C–O–C stretching (carbohydrates, polysaccharides) slight reductions in polysaccharide-associated peaks (C–O–C) may indicate involvement of carbohydrate moieties in biosorption. Emergence or enhancement of bands in the 600–800 cm⁻¹ region could suggest the formation of metal–oxygen coordination bonds, typical in biosorption processes. Cs^+^ and Sr^2+^ ions -loaded biomass resulted in appearance of three peaks at 3279, 2854, and 1455 cm^−1^. These spectral changes confirm that metal ions are effectively interacting with functional groups on the fungal biomass, mainly involving hydroxyl, amine, carbonyl, and possibly carboxylic groups. The comparative FTIR analysis clearly demonstrates the biosorption capability of *Aspergillus flavus* biomass for Cs and Sr ions. Shifts in peak positions and intensities confirm the involvement of functional groups such as O–H, N–H, C = O, and C–O in metal binding. This supports the potential application of *A. flavus* in the biosorption of radioactive or heavy metal-contaminated environments.

#### Thermal analysis

Thermal analysis of *Aspergillus flavus* biomass revealed four stages of degradation, as reported in Fig. 1**(B).** The initial stage exhibited a weight loss of 16.075% due to the desorption of water molecules from the biomass’s external surface, occurring within a temperature range of 32 °C to 213 °C. The second stage ranges from 213 °C to 438 °C with partial weight loss of 40.43%, which is assigned to the degradation of the total lipids^41^. The third stage ranges from 438 °C to 500 °C with a partial weight loss of 10.169%, which could belong to the decomposition of the polysaccharide structure. The fourth stage ranges from 500 to 600 °C with partial weight loss of 18.97%^42^.

#### SEM/EDS

SEM micrographs and EDS spectra of *Aspergillus flavus* biomass are presented in Fig. 1**(C).** Apparent morphology was seen as a smooth surface with porous cavities; The EDS spectrum showed the presence of C and O peaks along Fig. 1**(D)**. The presence of cell wall components in *Aspergillus flavus* biomass was probably the cause of the observed C and O peaks^40^.

#### Surface area

The nitrogen gas adsorption/desorption technique on *Aspergillus flavus* biomass has been utilized to determine the surface area. The total pore volume of *Aspergillus flavus* biomass is 0.0015 cm^3^ g^-1^. Brunauer–Emmett–Teller (BET) surface area and average pore radius of the *Aspergillus flavus* biomass material are 9.65 ms g^-1^ and 18.55 Å, respectively. Based on these characterizations, it can be deduced that the ability of *Aspergillus flavus* biomass to act as a biosorbent material. Thus, the assessment of *Aspergillus flavus* biomass material sorption characteristics is measured as described in the following section.

#### Chemical stability

The chemical stability (% solubility) of *Aspergillus flavus* biosorbent was assessed at room temperature, with the results presented in Table 1. The results indicate that *Aspergillus flavus* biosorbent exhibited stability in deionized distilled water, mineral acids, and alkalis. Table 1 indicates that the *Aspergillus flavus* biosorbent has comparatively excellent chemical stability when assessed against various sorbents.

**Table 1:** Chemical stability of *Aspergillus flavus* sorbent in various solvents.

Solvents	% Solubility
DDW	**N.D.**
0.1 M HCl	**0.004**
1 M HCl	**0.007**
0.1 M H_2_SO_4_	**0.006**
1 M H_2_SO_4_	**0.01**
0.1 M HNO_3_	**0.002**
1 M HNO_3_	**0.005**
0.1 M NaOH	**0.22**
1 M NaOH	**2.71**

*N.D. Not detected.

### Sorption properties of *Aspergillus flavus biomass*

#### Effect of pH

**Fig. 2:**
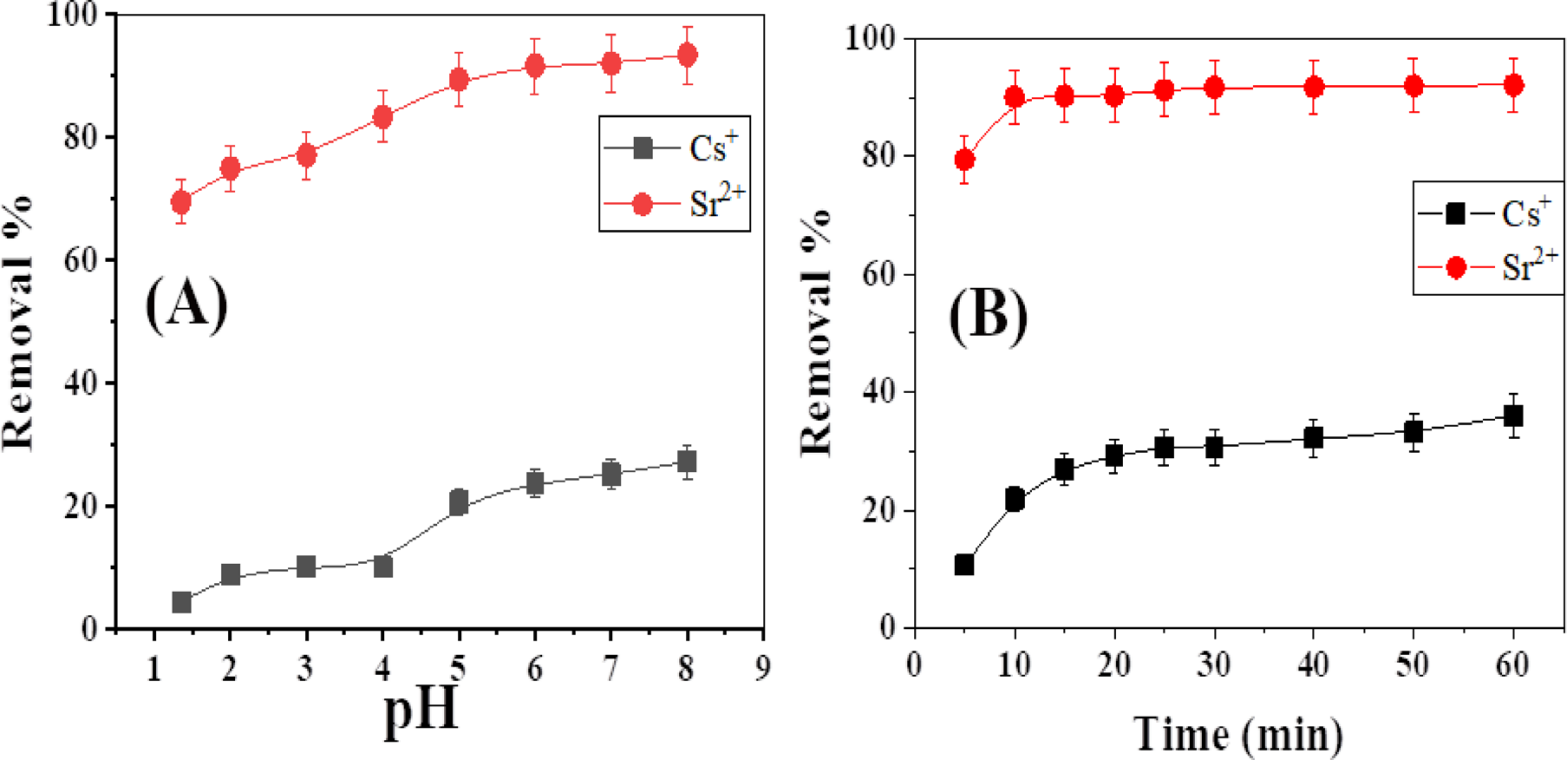
(**A**) Effect of pH, (**B**) Effect of contact time on sorption of Cs^+^ and Sr^2+^ onto *Aspergillus flavus*, m: 0.02 g, V: 0.1 L, pH: 5, T: 20.1 °C.

Multiple tests were conducted to evaluate the pH effect on metal removal by *Aspergillus flavus* biomaterial across different pH levels, as illustrated in Fig. 2(A). The study investigated the influence of initial pH on the removal of Cs^+^ and Sr^2+^ ions by *Aspergillus flavus*, utilizing initial ion concentrations of 50 mg/L, at time 24 h, and a temperature of 25 ± 0.1 °C. The pH of the solution is directly correlated with the quantity of adsorbed ions. An increase in pH values results in a higher concentration of adsorbed ions.

The pH of the sorption medium influences the metal ion’s solubility, and the ionization state of the carboxylate, phosphate, and amino groups present on the cell wall of fungi^43^. Hydrogen and metal ions compete for binding sites, affecting metal ion biosorption. The high proton concentration at lower pH increases the positive charge density on binding sites, reducing metal ion biosorption. With rising pH, the negative charge density on the cell surface increases as the metal binding sites undergo deprotonation. Subsequently, the metal ions improve their competitiveness for the limited binding sites, hence enhancing biosorption^44,45^.

A significant rise in the percentage of Sr^2+^ ion biosorption was found at pH 5.0, reaching around ~ 90%. The significant reliance of Sr^2+^ ion biosorption on pH indicates that surface complexation is the dominant sorption mechanism over cation exchange^30^. For Cs^+^ ions, the highest level of biosorption was seen at pH 8, with a sorption 27%. Consequently, pH 5 was identified as the optimal pH for subsequent experiments.

#### Effect of contact time

Figure 2(B) demonstrates the impact of contact time on the adsorption of 100 mg/L Cs^+^ and Sr^2+^ ions onto *Aspergillus flavus*. The percentage of the prepared biomaterial uptake grew as the contact time increased until it achieved equilibrium. We observed the highest uptake percentage for all the examined ions at 15 min, which indicates that the rate of Cs^+^ and Sr^2+^ adsorption onto *Aspergillus flavus* is high. The sorption equilibrium is highest for Sr^2+^ ions, with a sorption percentage of 90%. Cs⁺ ions have a lower sorption equilibrium, with a percentage of 27%. The active sites on the adsorbent’s surface contribute significantly to the absorption percentage. The greater sorption of Sr^2+^ compared to Cs^+^ on the *Aspergillus flavus* surface is primarily due to the higher charge density, smaller ionic radius, and stronger complexation ability of Sr^2+^ with the functional groups on the *Aspergillus flavus* surface. These factors lead to more effective ion exchange and surface complexation for Sr^2+^, resulting in higher sorption than Cs^+^. For ion exchange adsorption of Cs^+^ and Sr^2+^, the potential of the cation in solution (i.e., the ratio of charge to the radius of the hydrated ion, Z/r) determines the interaction force between the adsorbent and ions. Table 2 shows the hydration radius and potential of ions. Thus, the order of selectivity for Sr^2+^ is greater than Cs^+46^.

**Table 2:** The hydration radius and potential of the ions^46^.

Ion	Ionic Hydration Radius/Å	Electron Charge/Z	Potential/(Z/*r*)
Cs^+^	3.29	1	0.304
Sr^2+^	4.12	2	0.485

#### Kinetic isotherm models

The resulting data were utilized to investigate the step that determines the rate of the sorption process. Equations (4) and (5) represent the pseudo-1st and pseudo-2nd order equations, respectively.

4 $$Ln({q_e} - {q_t})=Ln{q_e} - \frac{{{K_1}}}{{2.303}}t$$

5 $$\frac{t}{{{q_t}}}=\frac{1}{{{K_2}q_{e}^{2}}}+\frac{1}{{{q_e}}}t$$.

**Fig. 3:**
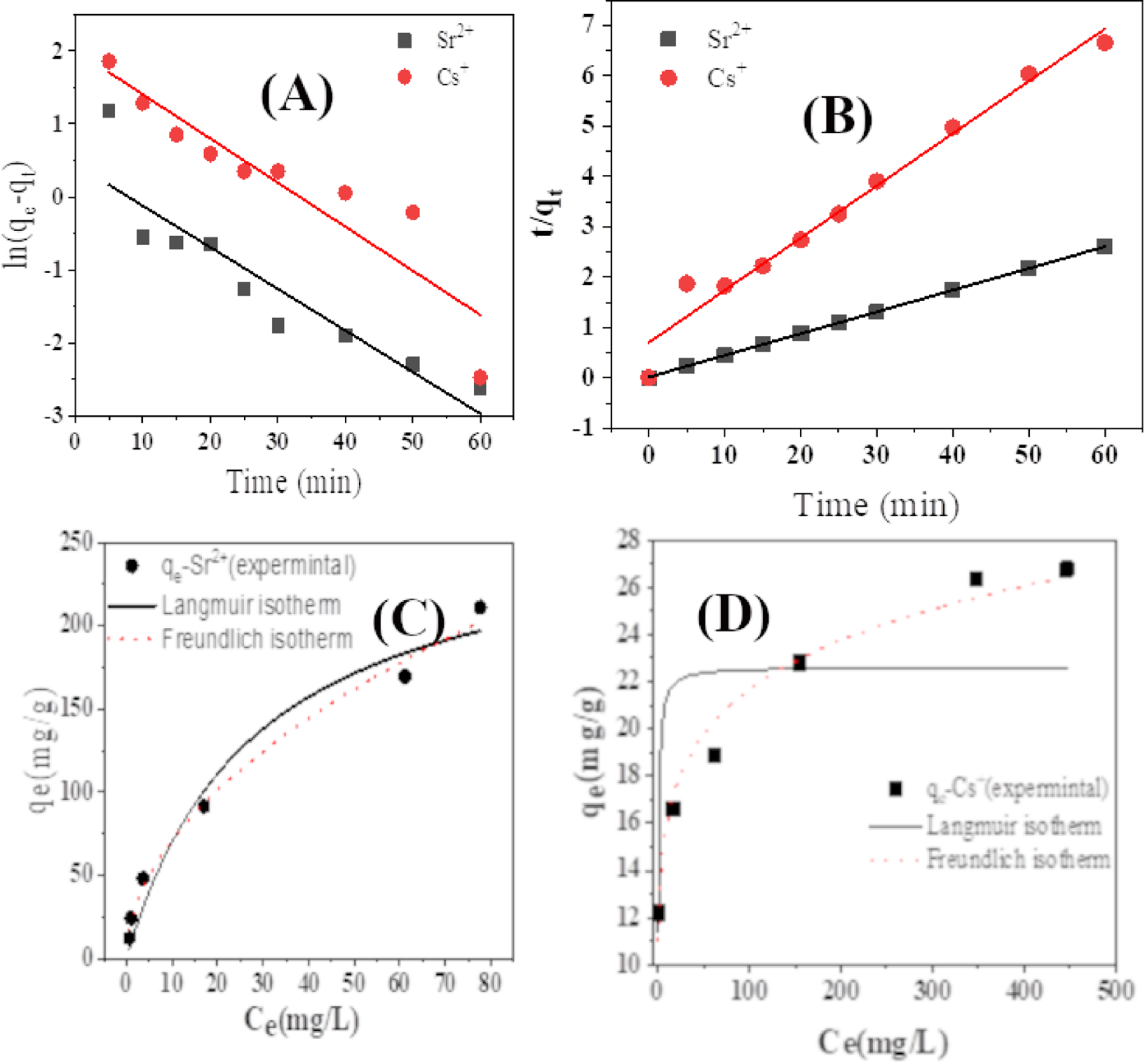
Plots of (**A**) pseudo-1st order and (**B**) pseudo-2nd order kinetic models for sorption of Cs^+^ and Sr^2+^ ions, and Nonlinear Langmuir and Freundlich isotherm plots for removing (**C**) Sr^2+^ and (**D**) Cs^+^ onto the *Aspergillus flavus*.

The variable q_e_ represents the quantity of metal ions adsorbed at equilibrium. The rate constants for the pseudo-1st and pseudo-2nd order equations are denoted as K_1_ and K_2_, respectively. The pseudo-1st order model, represented by Eq. (4), exhibits a linear relationship when plotting the logarithm of the difference between q_e_ and q_t_ against time, as shown in Fig. 3**(A)**. The determined value of the quantity of metal ions sorbed, q_e_ (calc.), was obtained from the intercept. However, it does not align with the experimental value, q_e_ (exp.) presented in Table 3. This indicates that the pseudo-1st order kinetic model is unsuitable for the sorption of Cs^+^ and Sr^2+^ ions onto biomaterial.

The sorption of Cs^+^ and Sr^2+^ ions onto the biomaterial was examined using a pseudo-second-order model. This was done by charting the ratio of the amount of sorbed ions to the time elapsed (t/q_t_) against time (t), as described by Eq. (5). The resulting plots showed straight lines, as illustrated in Fig. 3**(B)**. The q_e_ (calc.) values were determined from the slope and closely matched the experimental values, q_e_ (exp.). Table 3 shows the strong correlation coefficients achieved (R^2^ > 0.99) by applying the pseudo-second-order kinetic model. Hence, the pseudo-second-order kinetic model accurately corresponds to the experimental findings throughout the sorption process.

**Table 3:** Kinetic parameters of pseudo-1st order, pseudo-2nd order kinetic models, and parameters for Langmuir and Freundlich isotherm models used for the sorption of cs+^+^ and Sr^+2^ onto the biomaterial.

Adsorbed ion	q_e_ exp., mg⋅g^−1^	Pseudo-first-order			Pseudo-second-order		
		q_e_ calc., mg⋅g^−1^	k_1_, min^−1^	*R* ^2^	q_e_ calc., mg⋅g^−1^	k_2_, g⋅mg^−1^ ⋅min^−1^	*R* ^2^
Cs^+^	**9.02**	**7.45**	**−0.00101**	**0.83**	**9.64**	**0.015**	**0.97**
Sr^2+^	**23.03**	**1.57**	**−0.00095**	**0.81**	**23.12**	**0.111**	**0.99**
Adsorbed ion	q_e_ exp., mg⋅g^−1^	Langmuir model			Freundlich model		
		**q**_**max**_, **mg.g**^**−1**^	**K**_**L**_ **(L/mg)**	**R** ^**2**^	**K**_**F**_ **(mg**^**(1−n)**^ **Ln g**^**−1**^**)**	**n**	**R** ^**2**^
Cs^+^	**26.7**	**22.63**	**1.66**	**0.46**	**11.78**	**7.75**	**0.97**
Sr^2+^	**211.1**	**268.99**	**0.035**	**0.96**	**21.76**	**1.95**	**0.99**

#### Effect of initial concentration

The equilibrium tests were performed to investigate the sorption of Cs^+^ and Sr^2+^ utilizing biomaterial at varying initial concentrations of Cs^+^ and Sr^2+^ (50 to 500 mg/L) under ideal conditions. Their distribution between the liquid and solid phases dictates the equilibrium state in the extraction of metal ions. The equilibrium data were examined using a nonlinear approach, specifically models developed by Langmuir and Freundlich. The isotherm of Langmuir isotherm is an essential model for precisely elucidating physical and chemical sorption phenomena. The Langmuir theory argues that sorption occurs at distinct homogeneous sites. Equation (6)^47^ shows the mathematical expression for this model’s nonlinear version.

6 $${q_e}=\frac{{{K_L}{q_{\hbox{max} }}{C_e}}}{{(1+{K_L}{C_e})}}$$.

The variable q_e_ represents the concentration of metal ions on the sorbent at balance, measured in milligrams per gram. C_e_ represents the metal ions concentration in the solution at equilibrium, measured in mg/L. q_max_ is the maximum amount of metal ions the sorbent can adsorb per gram, measured in milligrams per gram. K_L_ is the Langmuir adsorption constant, measured in liters per milligram.

The second isotherm model is the Freundlich isotherm model. It states the existence of a heterogeneous adsorption surface with active spots exhibiting various energy levels. The Freundlich model is expressed by the Eq. (7)^48^,

7 $${q_e}={K_f}C_{e}^{{1/n}}$$.

where K_F_ (mg^1−n^ Ln g^−1^) is the Freundlich constant, which is influenced by the amount of the sorbent, and 1/n represents the affinity of the binding sites. The findings from the isotherm studies are displayed in Fig. 3**(C**,** D)** and Table 3. Figure 3**(C**,** D)** displays the fitting curves of the isotherms, indicating that the Freundlich isotherm model is the most appropriate for the adsorptive removal of Cs^+^ and Sr^+2^ using biomaterial.

#### Sorption thermodynamics

**Fig. 4:**
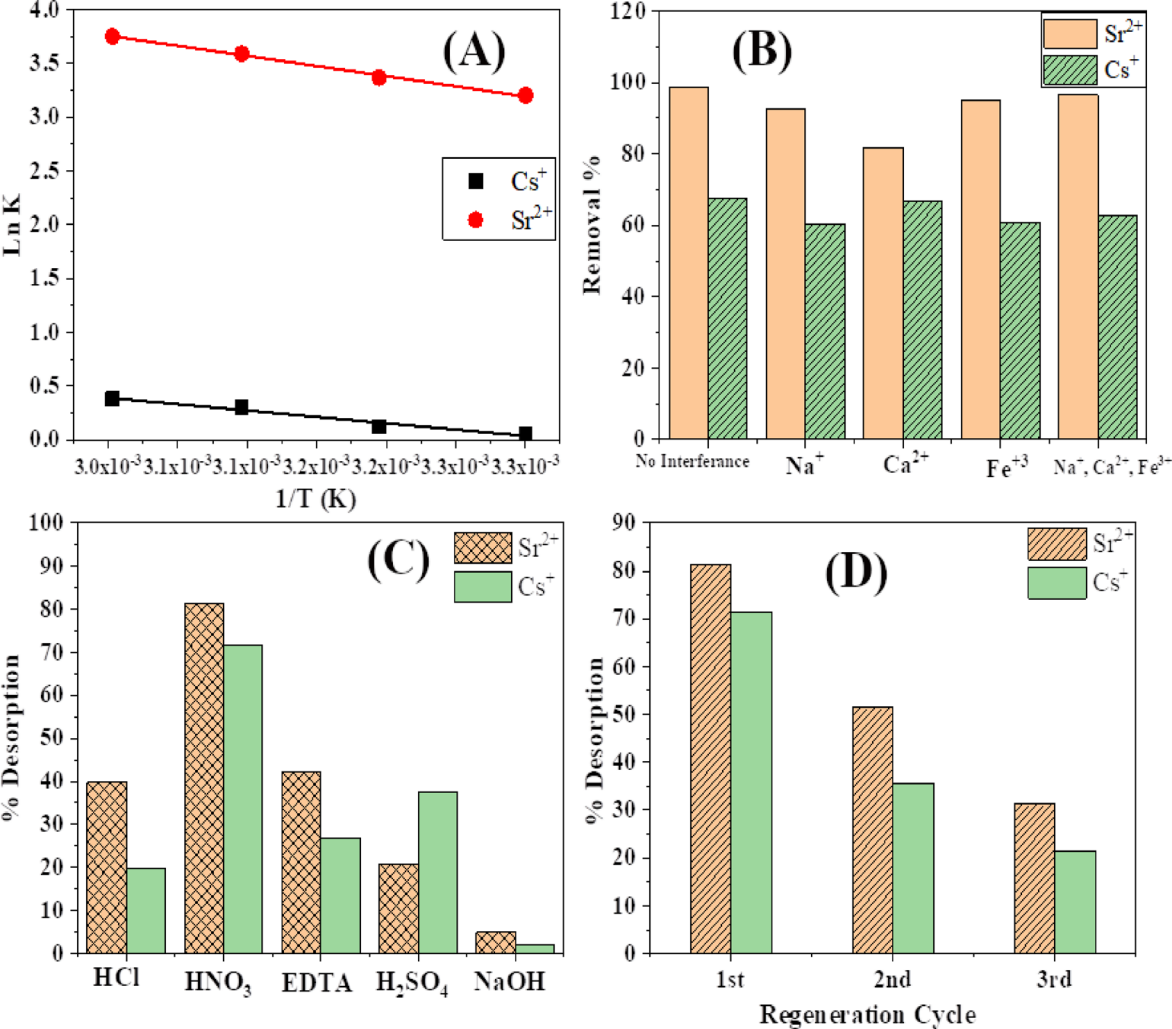
(**A**) Ln K_L_ Plot versus 1/T for removal of Cs^+^ and Sr^2+^, (**B**) impact of ions that interfere on Cs^+^, and Sr^2+^ removal (initial concentration of Cs^+^, and Sr^2+^: 25 mg/L. V/M = 2, pH = 5), (**C**) Recovery of Sr^2+^ and Cs^+^, (**D**) Reusability studies of *Aspergillus flavus*.

The current system’s thermodynamic properties were determined to better understand the biosorption process’s thermodynamics. The change in Gibbs free energy (ΔG) determines randomness. The reaction occurs spontaneously if ΔG is negative at a specific temperature. The Van’t Hoff equation was utilized to calculate the thermodynamic parameters as follows:

8 $$\Delta G= - RT\ln {K_L}$$

9 $$\ln {K_L}=\frac{{\Delta S}}{R} - \frac{{\Delta H}}{{RT}}$$.

where R = the universal gas constant, T = the system absolute temperature (K), and K_L_ = the standard thermodynamic balance constant (L/g) defined by q_e_/C_e_. Figure 4**(A)** illustrates the values of ΔH and ΔS used to calculate from the slope and intercept of a graph of Ln K_L_ vs. 1/T, and the results are shown in Table 4.

**Table 4:** Thermodynamic parameters for cs+^+^ and Sr^2+^ sorption on *Aspergillus flavus*.

Adsorbent	Temp (K)	K_L_	ΔG (K.J.mol^−1^)	ΔH (K.J.mol^−1^)	ΔS (J.K^−1^mol^−1^)
*Aspergillus flavus*/Sr^2+^	303	24.6	−8.07	15.67	78.26
	313	29.1	−8.77		
	323	36.3	−9.64		
	333	42.6	−10.39		
*Aspergillus flavus*/Cs^+^	303	1.1	−0.24	9.82	32.70
	313	1.1	−0.25		
	323	1.4	−0.90		
	333	1.5	−1.12		

The ΔG values for Cs^+^ and Sr^2+^ ions were consistently negative at all temperatures. This suggests that the sorption processes occurred spontaneously. Moreover, the change in ΔG exhibited an upward trend as the temperature decreased, indicating that temperature played a significant role in the sorption process^49^. The fact that ΔH is positive suggests that *Aspergillus flavus*’s ion sorption was endothermic because it involved heat absorption. The ΔH values for Cs^+^ and Sr^2+^ metal ions indicate that the sorption of these metals onto *Aspergillus flavus* was physisorption, as the values did not exceed 40 KJ/mol. Furthermore, the positive ΔS value indicates an increase in randomness at the interface between *Aspergillus flavus* and the solution, as shown in Table 3.

#### Effect of interfering ions

We examined the impact of coexisting ions on removing Cs^+^ and Sr^2+^ by *Aspergillus flavus* by individually and collectively adding 25 mg/L of Na^+^, Ca^2+^, and Fe^3+^ ions to the solutions, as illustrated in Fig. 4**(B)**. The impact of these ions on the mixture of Cs^+^ and Sr^2+^ was subsequently examined. We calculated the elimination percentage to evaluate the selectivity for Cs^+^ and Sr^2+^. The percentage removal of Cs^+^ diminished correspondingly with the concentration of the monovalent cation, Na^+^. Divalent cations, Ca^2+^, had a diminished effect on the removal percentage of Cs^+^. Unlike Cs^+^ and Sr^2+^, the divalent Ca^2+^ cation significantly influenced adsorption. Specifically, Ca^2+^, with chemical characteristics equivalent to those of Sr^2+^, significantly impeded Sr^2+^ adsorption. Meanwhile, Sr^2+^ adsorption was minimally influenced by monovalent cations. The competitive influence of cations on the adsorption of Cs^+^ and Sr^2+^ adhered to the sequence Na^+^ > Fe^3+^ > Ca^2+^ for Cs^+^ and Ca^2+^ > Na^+^ > Fe^3+^ for Sr^2+^.

#### Biosorbent desorption and reusability

The desorption process involves removing the adsorbate from the biosorbent surface, making the biosorbent material reusable in the treatment process. This work involved the desorption of Sr^2+^ and Cs^+^ from the *Aspergillus flavus* biosorbent surface, which had been previously loaded with these metal ions, utilizing a batch approach with 0.1 M various eluting agents, including H_2_SO_4_, HCl, HNO_3_, NaOH, and EDTA. The results demonstrated that HNO_3_ (0.1 M) is the most effective, achieving desorption percentages of 81.2% for Sr^2+^ and 71.5% for Cs^+^ Fig. 4**(C)**.

The reusability of the biosorbent is a crucial factor influencing the biosorption system’s economic feasibility and minimizing the operational costs of the separation process. Figure 4**(D)** depicts the outcomes of the reusability investigations of *Aspergillus flavus* biosorbents. The biosorbent was regenerated using HNO_3_ (0.1 M) and reused efficiently for three cycles. A significant reduction in biosorption capacity (regenerated with HNO_3_, 0.1 N) was seen after more than three cycles, attributed to the decreased surface functional groups resulting from chemical regeneration and ongoing saturation of binding spots^50,51^.

#### Suggest a biosorption mechanism

The removal of metal ions by fungal biomass occurs through three mechanisms: **chemical transformations**^52^**bioaccumulation**^9,23,30^and **biosorption**^53^.

**Fig. 5:**
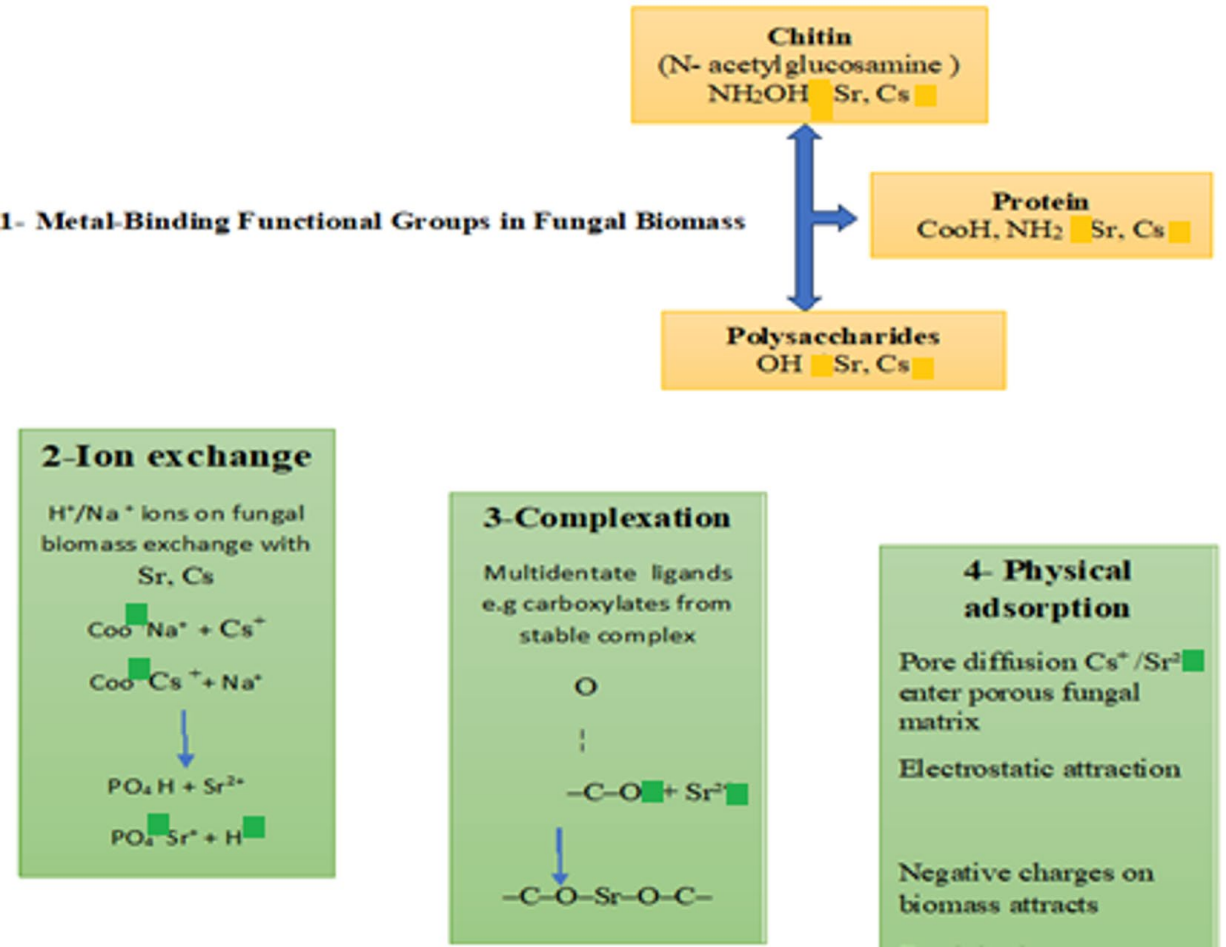
Diagram illustrating the biosorption of cesium and strontium from aqueous solution by A flavus biomass.

This study focuses on biosorption by *Aspergillus flavus* dead biomass, which exhibits enhanced metal uptake capacity^52^. The process involves **surface complexation**, where metal ions coordinate with electron-donating groups on the cell wall, forming stable chelates^53^ and ion exchange, where native cations are displaced by target metals, maintaining charge balance^53^. Cell wall adsorption dominates metal removal in dead biomass systems^54^. Figure 5 schematically illustrates these mechanisms for Cs^+^ and Sr^+2^ biosorption by *Aspergillus flavus*.

#### Comparison of sorption capacity

We compared our findings to those published in the literature to determine the prepared *Aspergillus flavus* biomass’s adsorption efficiency. The maximum adsorption capacity (q_max_) of various adsorbents for the adsorption of Cs^+^ and Sr^2+^ is shown in Table 5 from the literature. However, a direct comparison of the adsorption capacity between the synthesized *Aspergillus flavus* used in this study and other adsorbents reported in the literature is challenging due to differences in experimental conditions applied in those studies. The maximum adsorption capacity for Cs^+^ and Sr^2+^ ions in our study was determined to be 26.6 and 211.1 mg g^−1,^ respectively.

**Table 5:** Comparative sorption capacities of various adsorbents for Sr^2+^ and cs+^+^ from the liquid phase.

Sorbents	Adsorption capacity, mg⋅g^−1^		Adsorption conditions				Ref.
	Cs^+^	Sr^2+^	pH	Initial conc., (mg/L)	Time	Temp., °C	
Magnetic graphene oxide	NR^*^	9.29	4	10	48 h	20	^55^
zinc ferrite-humic acid composite	NR^*^	42.55	5	350	100–150 min	25	^56^
SiO_2_-Fe-CN nanomaterial	32.26	NR	5.5	500	60 min	55	^57^
Silica loaded with *Aspergillus brasiliensis* biomass	23.38	66.31	7	50	40 min	25	^58^
K-hexacyanoferrate-PVA	7	NR	1–12	150	60 min	20	^59^
Polycondensed feldspar	39.82	NR	6.5–7	50	180–240 min	50	^4^
Polycondensed perlite	37.0	NR					
Polycondensed feldspar/perlite blend	60.0	NR					
***Aspergillus flavus*** **biomass**	26.7	211.1	5	50	4 h	20	This work

*NR: not recorded.

## Conclusion

The dried biomass, ***Aspergillus flavus***, functioned as an effective, environmentally friendly, and adsorbent for remediation aqueous solutions containing Cs^+^ and Sr^2+^ ions. This fungus biosorbent exhibits significant stability in aqueous environments, including exposure to acids, and bases, particularly at low concentrations, and possesses an irregular shape. It possesses commendable thermal stability. The fast adsorption of cesium and strontium, as demonstrated in the current biomass, is proposed to be significant as an effective biosorbent, allowing rapid sorption kinetics. The positive value of ΔH indicates the endothermic characteristic of the process. The measurement of ΔS indicated increased randomness at the adsorbate-adsorbent interface during the operation. The ΔH values are < 40 kJ/mol, indicating that the process was managed by physical biosorption. Upon treatment of the loaded biosorbent material with 0.1 M HNO_3_, all adsorbed metal ions were eluted, rendering the biosorbent devoid of any sorbed metal, thus enabling its efficient reuse for three cycles. The disposal of treated wastewater and biosorption sludge is a critical environmental consideration. While *Aspergillus flavus* biomass effectively removes Cs and Sr ions, the adsorbed radionuclides onto the used biomass materials must be managed in a later stage. To minimize environmental risks, the incineration of spent biomass, ensuring volume reduction, is conducted in licensed radioactive waste incineration, mitigating airborne releases through HEPA filtration, and remote handling and shielded storage are mandatory to comply with ALARA principles. The encapsulation or solidification methods can be employed for the resulting ash to stabilize the radioactive aqueous solution before disposal in designated waste storage facilities. Encapsulating the sludge and storing it in deep underground facilities designed for long-term containment of radioactive materials. Future studies should explore regeneration techniques for the fungal biomass to reduce waste generation and enhance cost-effectiveness, with details of safe handling of waste management.

## Data Availability

The datasets used and/or analysed during the current study available from the corresponding author on reasonable request.
